# Emerging types of Shiga toxin-producing *E. coli* (STEC) O178 present in cattle, deer, and humans from Argentina and Germany

**DOI:** 10.3389/fcimb.2014.00078

**Published:** 2014-06-17

**Authors:** Angelika Miko, Marta Rivas, Adriana Bentancor, Sabine Delannoy, Patrick Fach, Lothar Beutin

**Affiliations:** ^1^Division of Microbial Toxins, National Reference Laboratory for Escherichia coli, Federal Institute for Risk Assessment (BfR)Berlin, Germany; ^2^Servicio Fisiopatogenia, Departamento de Bacteriología, Instituto Nacional de Enfermedades Infecciosas-ANLIS “Dr. Carlos G. Malbrán”Buenos Aires, Argentina; ^3^Cátedra de Microbiología, Facultad de Ciencias Veterinarias, Universidad de Buenos AiresBuenos Aires, Argentina; ^4^Food Safety Laboratory, French Agency for Food, Environmental and Occupational Health (Anses)Maisons-Alfort, France

**Keywords:** *E. coli* O178, STEC, Shiga toxins, virulence, genotyping, PFGE, real-time PCR micro array

## Abstract

More than 400 serotypes of Shiga toxin-producing *Escherichia coli* (STEC) have been implicated in outbreaks and sporadic human diseases. In recent years STEC strains belonging to serogroup O178 have been commonly isolated from cattle and food of bovine origin in South America and Europe. In order to explore the significance of these STEC strains as potential human pathogens, 74 German and Argentinean *E. coli* O178 strains from animals, food and humans were characterized phenotypically and investigated for their serotypes, *stx*-genotypes and 43 virulence-associated markers by a real-time PCR-microarray. The majority (*n* = 66) of the O178 strains belonged to serotype O178:H19. The remaining strains divided into O178:H7 (*n* = 6), O178:H10 (*n* = 1), and O178:H16 (*n* = 1). STEC O178:H19 strains were mainly isolated from cattle and food of bovine origin, but one strain was from a patient with hemolytic uremic syndrome (HUS). Genotyping of the STEC O178:H19 strains by pulsed-field gel electrophoresis revealed two major clusters of genetically highly related strains which differ in their *stx*-genotypes and non-Stx putative virulence traits, including adhesins, toxins, and serine-proteases. Cluster A-strains including the HUS-strain (*n* = 35) carried genes associated with severe disease in humans (*stx*2a, *stx*2d, *ehxA*, *saa*, *subAB1*, *lpfA_O113_*, *terE* combined with *stx*1a, *espP*, *iha*). Cluster B-strains (*n* = 26) showed a limited repertoire of virulence genes (*stx*2c, *pagC*, *lpfA_O113_*, *espP*, *iha*). Among O178:H7 strains isolated from deer meat and patients with uncomplicated disease a new STEC variant was detected that is associated with the genotype *stx*1c/*stx*2b/*ehxA*/*subAB2*/*espI*/*[terE]*/*espP*/*iha*. None of the STEC O178 strains was positive for locus of enterocyte effacement (LEE)- and *nle*-genes. Results indicate that STEC O178:H19 strains belong to the growing group of LEE-negative STEC that should be considered with respect to their potential to cause diseases in humans.

## Introduction

Shiga toxin-producing *Escherichia coli* (STEC) is a zoonotic pathogen of significant public health concern. Worldwide, STEC has been associated with both outbreaks and sporadic cases of human disease, ranging from mild diarrhea to hemorrhagic colitis (HC) and life-threatening hemolytic uremic syndrome (HUS) (Melton-Celsa et al., 2012). In Argentina, HUS is endemic with 500 cases per year and an incidence of 17/100,000 children less than 5 years of age (Rivas et al., 2010). In contrast, Germany notifies about 65 HUS cases per year and an incidence of 0.1/100,000 population (Robert Koch-Institut, 2011). STEC strains produce one or both of two major types of potent cytotoxins called Shiga toxins Stx1 and Stx2, which function as primary virulence factors in human disease. Stx1 and Stx2 are immunologically not cross-reactive and display 57 and 60% nucleotide sequence identity in their A and B subunits, respectively (Muthing et al., 2009). The more homogenous Stx1 family consists of Stx1a, Stx1c, and Stx1d. The more heterogeneous Stx2 family comprises Stx2a and the variants designated Stx2b, Stx2c, Stx2d, Stx2e, Stx2f, and Stx2g (Scheutz et al., 2012). Stx2d differs from all known Stx types because it is activated in its biological activity by elastase, a constituent of the intestinal mucus (Melton-Celsa et al., 1996).

The STEC group is serologically highly diverse. More than 400 O:H types of STEC associated with infections in humans were recorded in 2005 (Scheutz and Strockbine, 2005). In the last years, the *E. coli* serotyping scheme was extended due to the emergence of STEC strains belonging to the newly defined O-serogroups O174 to O181 (Scheutz et al., 2004). Cattle have been recognized as the most important animal reservoir of STEC causing disease in humans (Hussein, 2007). However, there are only few data available about animal reservoir, epidemiology and human pathogenicity of the new emerging STEC types. STEC O178 was first described in 2004; the prototype strain (O178:H7, *stx1*, and *stx2*) was isolated from raw meat (Scheutz et al., 2004). STEC O178:H19 strains were frequently isolated from beef in Germany (Beutin et al., 2007; Werber et al., 2008; Slanec et al., 2009) and Argentina (Sanz et al., 2007; Kruger et al., 2011). Cattle were identified as important reservoir for STEC O178:H19 in South America (Cergole-Novella et al., 2007; Oliveira et al., 2008; Fernandez et al., 2010; Kruger et al., 2011; Masana et al., 2011; Tanaro et al., 2012) and in Europe (Blanco et al., 2004; Aidar-Ugrinovich et al., 2007; European Food Safety Authority and European Centre for Disease Prevention and Control, 2012). Sporadic cases of human infections with STEC O178:H19 were reported from Germany (Bielaszewska et al., 2006; Werber et al., 2008) and Brazil (De Toni et al., 2009). STEC O178:H19 strains were also reported as the cause of HUS cases in Belgium (Buvens et al., 2010) and Argentina (Giugno et al., 2007). Besides O178:H19, STEC O178:H7 producing *stx*2f were associated with diarrhea in humans (Prager et al., 2009).

At present, relatively few cases of human illness were associated with STEC O178 infections. Such cases might be underreported because serotyping of non-O157 STEC is only performed by a small number of reference laboratories worldwide (Bettelheim, 2007). On the other hand, it is possible that STEC O178 strains are less virulent for humans compared with other frequently isolated STEC types. A number of factors were shown to play a role in the virulence of STEC for humans. Some variants of the Stx2 gene, namely Stx2a, Stx2c and the mucus activatable Stx2d were significantly associated with HC and HUS in human patients (Friedrich et al., 2002; Jelacic et al., 2003; Bielaszewska et al., 2006). Besides production of Shiga toxins, the attaching and effacing phenotype encoded by the genomic locus of enterocyte effacement (LEE), and the presence of some non-LEE genomic O-islands (OI) such as OI-122, OI-71 and OI-57, are significantly associated with STEC types that are frequently involved in outbreaks and cause HC and HUS in humans (Karmali et al., 2003; Coombes et al., 2008; Konczy et al., 2008; Bugarel et al., 2010a,b). The LEE encodes for the outer-membrane adhesin intimin (*eae*), the translocated intimin receptor Tir, a type III secretion system, and effector proteins translocated by the secretion system. OI-122, OI-71 and OI-57 harbor a number of non-LEE encoded effector (*nle*) genes which encode potential virulence traits (Coombes et al., 2008; Konczy et al., 2008). Additional adhesins and putative virulence factors encoded outside of the LEE in genomic islands or on large plasmids, such as pO157 in LEE-positive strains and pO113 in LEE-negative strains, may play a role in pathogenesis. Plasmids pO157 and pO113 share several virulence genes such as *ehx*A, which encodes an EHEC enterohemolysin (Schmidt et al., 1995), *espP*, which encodes an extracellular serine protease (Brunder et al., 1999), and *iha* which was also found within a genomic island, and encodes an adherence-conferring protein similar to *Vibrio cholerae* IrgA (Tarr et al., 2000; Schmidt et al., 2001; Newton et al., 2009). However, pO113 lacked the pO157-encoded type II secretion system (*etpD*), a periplasmic catalase peroxidase (*katP*) (Schmidt et al., 1997; Brunder et al., 1999) and a homolog of the adherence-promoting protein ToxB *(toxB*) (Tatsuno et al., 2001). On the other hand, pO113 encodes a number of unique virulence-associated determinants, including the autoagglutinating adhesin Saa (*saa*) and the subtilase-like serine protease toxin SubAB (*subAB*) (Paton et al., 2004). Saa encoded by LEE-negative O113:H21 (Paton et al., 2001) and O91:H21 (Paton and Paton, 2002) strains was characterized as putative colonization factor that enables LEE-negative STEC strains to adhere to the intestinal epithelium of humans. SubAB might also contribute to the virulence of LEE-negative STEC strains in humans by a synergistic action with Stx (Paton et al., 2004). Furthermore, long polar fimbriae (LPF) (*lpfA*) closely related to LPF of *Salmonella enterica* serovar Typhimurium, were proposed to be novel adhesion factors (Doughty et al., 2002; Torres et al., 2002).

Due to the recent emergence of STEC O178 strains in cattle and food of bovine origin on both the South American and the European continent, we became interested in studying the virulence potential of such strains. For this, we compared *E. coli* O178 isolates from Argentina and Germany originating from animals, food and humans in regard to their virulence properties, including toxins, adhesins, and serine proteases. Furthermore, we analyzed the genetic relationships among these strains using data obtained by pulsed-field gel electrophoresis. Our results indicate that STEC O178:H19 belong to the growing group of LEE-negative strains that are able to cause severe illness in humans (Newton et al., 2009).

## Materials and methods

### Bacterial strains

A total of 74 *E. coli* serogroup O178 strains were characterized in this study (Table 1). All except three were isolated between 2005 and 2012 and originated from Argentina (*n* = 49) or Germany (*n* = 25). Forty-two of the strains were isolated from feces or carcasses of cattle originating from different farms and 19 from food, such as beef (*n* = 4), ground beef (*n* = 12), ground beef/pork (*n* = 2), and raw cows milk (*n* = 1). Six strains were obtained from other food sources, such as ground lamb (*n* = 1), red deer meat (*n* = 4), and salad (*n* = 1). Of the four strains from humans examined, three were isolated from patients with uncomplicated disease and one from a HUS patient. Three strains were from rectal swabs of dogs.

**Table 1:** **Characteristic traits of the *E. coli* O178 isolates investigated in this study**.

						**Presence of (putative) virulence genes^a^**									
**Strain**	**H-type^b^**	**Source**	**Origin**	**Year**	***stx*-genotype^c^**	***ehxA***	***espP***	***iha***	***saa***	***subAB^d^***	***tia***	***lpfA_O113_***	***terE***	***pagC***	***espI^e^***
CB10181	19	beef	Germany	2005	*stx*1a *stx*2a	+	+	+	+	−	nd	+	−	−	−
CB10542	19	ground beef	Germany	2006	*stx*1a *stx*2a *stx*2c	+	+	+	+	−	nd	+	−	−	−
CB10545	19	ground beef	Germany	2006	*stx*2c	−	+	+	−	−	nd	+	−	+	−
CB10659	19	ground lamb	Germany	2006	*stx*2a	+	+	+	+	*subAB1*	−	+	−	+	−
CB10751	19	meat, deer	Germany	2007	*stx*2a	+	+	+	+	*subAB1*	−	+	−	+	−
CB10836	19	ground beef/pork	Germany	2007	*stx*2c	−	+	+	−	−	nd	+	−	+	−
CB11017	19	ground beef	Germany	2007	*stx*1a *stx*2a	+	+	+	+	*subAB1*	−	+	−	−	−
CB11536	19	ground beef/pork	Germany	2008	*stx*1a *stx*2a	+	+	+	+	−	nd	+	+	−	−
CB11726	19	ground beef	Germany	2008	*stx*2a *stx*2d	+	+	+	+	*subAB1*	−	+	−	+	−
CB12008	19	raw milk	Germany	2009	*stx*1a *stx*2a *stx*2d	+	+	+	+	*subAB1*	−	+	+	−	−
CB12145	19	ground beef	Germany	2009	*stx*1a *stx*2a	+	+	+	+	−	nd	+	+	−	−
CB12220	19	beef	Germany	2009	*stx*1a *stx*2a	+	+	+	+	*subAB1*	−	+	+	−	−
CB13785	19	beef	Germany	2011	*stx*1a *stx*2d	+	+	+	+	−	nd	+	−	−	−
CB13787	19	beef	Germany	2011	*stx*1a *stx*2d	+	+	+	+	−	nd	+	−	−	−
CB14014	19	ground beef	Germany	2012	*stx*1a *stx*2a	+	+	+	+	−	nd	+	+	−	−
CB14018	19	cattle	Germany	2012	*stx*2c	−	+	+	−	−	nd	+	−	+	−
CB14051	19	cattle	Germany	2012	*stx*1a *stx*2d	+	+	+	+	−	nd	+	−	−	−
188/06 30	19	cattle	Argentina	2006	*stx*1a *stx*2a	+	+	+	+	−	nd	+	+	−	−
276	19	dog	Argentina	2005	*stx*2c	−	+	+	−	−	nd	+	−	+	−
377ii	19	dog	Argentina	2005	*stx*2c	−	+	+	−	−	nd	+	−	+	−
489/06 16	19	cattle	Argentina	2006	*stx*2c	−	+	+	−	−	nd	+	−	+	−
489/06 24	19	cattle	Argentina	2006	*stx*1a *stx*2a	+	+	+	+	−	nd	+	+	−	−
489/06 36	19	cattle	Argentina	2006	*stx*2c	−	+	+	−	−	nd	+	−	+	−
612/06 21	19	cattle	Argentina	2006	*stx*1a *stx*2a	+	+	+	+	−	nd	+	+	−	−
612/06 33	19	cattle	Argentina	2006	*stx*2c	−	+	+	−	−	nd	+	−	+	−
612/06 35	19	cattle	Argentina	2006	*stx*1a *stx*2a	+	+	+	+	−	nd	+	+	−	−
612/06 9	19	cattle	Argentina	2006	*stx*2c	−	+	+	−	−	nd	+	−	+	−
96/00	19	human (HUS)	Argentina	2000	*stx*1a *stx*2a	+	+	+	+	*subAB1*	−	+	−	−	−
C11	19	ground beef	Argentina	2006	*stx*2c	−	+	+	−	−	nd	+	−	+	−
C18iii	19	ground beef	Argentina	2006	*stx*2c	−	+	+	−	−	nd	+	−	+	−
C22	19	ground beef	Argentina	2006	*stx*2c *stx*2b	−	+	+	−	−	nd	+	−	+	−
C27	19	ground beef	Argentina	2006	*stx*1a *stx*2d	+	+	+	+	*subAB1*	−	+	−	−	−
C39	19	ground beef	Argentina	2007	*stx*2c	−	+	+	−	−	nd	+	−	+	−
C42	19	ground beef	Argentina	2007	*stx*2c	−	+	+	−	−	nd	+	−	+	−
FP−001	19	cattle	Argentina	2006	*stx*2c	−	+	+	−	−	nd	+	−	+	−
FP−023	19	cattle	Argentina	2007	*stx*2c	−	+	+	−	−	nd	+	−	+	−
FP−024	19	cattle	Argentina	2007	*stx*1a *stx*2a	+	+	+	+	*subAB1*	−	+	−	−	−
FP−065	19	cattle	Argentina	2007	*stx*1a *stx*2a	+	+	+	+	−	nd	+	+	−	−
FP−067	19	cattle	Argentina	2007	*stx*1a *stx*2a	+	+	+	+	−	nd	+	+	−	−
FP−070	19	cattle	Argentina	2007	*stx*1a *stx*2a	+	+	+	+	*subAB1*	−	+	−	−	−
FP−073	19	cattle	Argentina	2007	*stx*1a *stx*2a	+	+	+	+	*subAB1*	−	+	−	−	−
FP−075	[19]	cattle	Argentina	2007	*stx*1a *stx*2a	+	+	+	+	*subAB1*	−	+	−	−	−
FP−080	19	cattle	Argentina	2007	*stx*2a *stx*2d	+	+	+	+	*subAB1*	−	−	−	−	−
FP−082	19	cattle	Argentina	2007	*stx*2c	−	+	+	−	−	nd	+	+	+	−
FP−087	19	cattle	Argentina	2007	*stx*1a *stx*2a	+	+	+	+	*subAB1*	−	+	−	−	−
FP−089	19	cattle	Argentina	2007	*stx*2c	−	+	+	−	−	nd	+	−	+	−
FP−090	19	cattle	Argentina	2007	*stx*2c	−	+	+	−	−	nd	+	+	+	−
FP−100	19	cattle	Argentina	2007	*stx*2a	+	+	+	+	*subAB1*	−	+	−	+	−
FP−110	19	cattle	Argentina	2007	*stx*1a *stx*2a	+	+	+	+	−	nd	+	+	−	−
FP−112	19	cattle	Argentina	2007	*stx*2c	−	+	+	−	−	nd	+	−	+	−
FP−113	19	cattle	Argentina	2009	*stx*2c	−	+	+	−	−	nd	+	−	+	−
FP−116	19	cattle	Argentina	2007	*stx*2c	−	+	+	−	−	nd	+	−	+	−
FP−134	19	cattle	Argentina	2007	*stx*2c	−	+	+	−	−	nd	+	−	+	−
FP−163	19	cattle	Argentina	2007	*stx*2c	−	+	+	−	−	nd	+	−	+	−
FP−194	19	cattle	Argentina	2008	*stx*2c	−	+	+	−	−	nd	+	+	+	−
FP−203	19	cattle	Argentina	2007	*stx*1a *stx*2a	+	+	+	+	−	nd	+	−	−	−
FP−229	[19]	cattle	Argentina	2008	*stx*2c	−	+	+	−	−	nd	+	−	+	−
FP−245	19	cattle	Argentina	2008	*stx*2c	−	+	+	−	−	nd	+	−	+	−
I−005	19	cattle	Argentina	2006	*stx*1a *stx*2a	+	+	+	+	*subAB1*	−	+	−	−	−
I−011	[19]	cattle	Argentina	2007	*stx*2c	−	+	+	−	−	nd	+	−	+	−
I−023	[19]	cattle	Argentina	2007	*stx*2c	−	+	+	−	−	nd	+	−	+	−
I−039	19	cattle	Argentina	2007	*stx*1a *stx*2a *stx*2d	+	+	+	+	*subAB1*	−	+	+	+	−
I−058	19	cattle	Argentina	2007	*stx*2c	−	+	+	−	−	nd	+	−	+	−
I−073	19	cattle	Argentina	2007	*stx*1a *stx*2a	+	+	+	+	−	nd	+	−	−	−
I−115	19	cattle	Argentina	2007	*stx*1a *stx*2a	+	+	+	+	*subAB1*	−	+	−	−	−
P5a	[19]	dog	Argentina	2007	*stx*2c	−	+	+	−	−	nd	+	−	+	−
CB06224	[7]	human (D)	Germany	1996	*stx*1c *stx*2b	+	+	+	−	*subAB2*	+	+	−	−	*+*
CB06571	[7]	human (AS)	Germany	1996	*stx*1c	+	+	+	−	*subAB2*	+	+	+	−	*+*
CB09017^f^	[7]	human (E)	Germany	2001	−	−	−	−	−	−	nd	+	−	−	−
CB10124	[7]	meat, deer	Germany	2005	*stx*1c *stx*2b	+	+	+	−	*subAB2*	+	+	−	−	*+*
CB11675	[7]	meat, deer	Germany	2008	*stx*1c *stx*2b	+	+	+	−	*subAB2*	+	+	+	−	*+*
CB12072	7	cattle	Germany	2009	−	−	−	−	−	−	nd	+	−	−	−
CB11890	10	meat, deer	Germany	2009	*stx*1a	−	+	+	−	*subAB1*	−	+	−	−	−
CB12372	16	salad	Germany	2009	−	−	−	−	−	−	nd	+	−	−	−

*HUS, hemolytic uremic syndrome; D, diarrhea; E, enteritis; AS, asymptomatic carrier; +, the gene is present; −, the gene is absent*.

a *The genes encode the following proteins: ehxa, enterohemolysin; espP, serine protease (EspP); iha; iron-regulated gene A homolog adhesin (Iha); saa, STEC autoagglunating adhesin (Saa); subAB, subtilase-like serine protease toxin (SubAB); tia, invasion determinant described in enterotoxigenic E. coli; lpfA_O113_, major subunit of long polar fimbriae of STEC O113; OI-43/48-terE (marker of ter cluster), tellurite resistance; OI-122-pagC (Z4321), homolog of PagC protein of Salmonella enterica serovar Typhimurium; espI, serine protease (EspI)*.

*Twenty eight of the LEE- or OI-encoded virulence-associated genes (eae, espG [LEE], ent, nleB, nleE, efa1, efa2 [OI-122], nleF, nleH1-2, nleA, nleG, espM1, ecs1822 [OI-71], ureD [OI-43/48] (marker für ure cluster), ecs1763, espN, espM2, espX2, espX7, espO1-1, espY4-2, espX6, espW, espY1, espY3, nleH1-1, nleD-2, and espJ) as well as the plasmid pO157-encoded genes katP, etpD, and toxB as well as the lpfA_O157_ gene which encodes the major subunit of long polar fimbriae of EHEC O157:H7 EDL933, and the EAEC marker gene aggr were not detected in any of the strains (except the EPEC O178:H7 strain CB09017, see below)*.

b *[] indicates nonmotile strains that were analyzed for their fliC genotypes by PCR/RFLP of HhaI-digested fliC PCR products*.

c *The standardized Stx nomenclature published by Scheutz et al. (2012) was used*.

d *Two variants of the subAB were detected; subAB1 indicates subAB_98NK2_, plasmid pO113-encoded. No tia gene was detected in these strains. subAB2 indicates subAB_ED32_, chromosomal encoded on a putative PAI. The tia gene was detected in these strains. nd, not done*.

e *Encoded by the locus of protease and adherence (LPA) which is located in the chromosome (Schmidt et al., 2001)*.

f *The EPEC O178:H7 strain CB09017 revealed genes eae, espG, and espF (LEE-encoded) and genes ent/espL2, nleB, nleE, efa1, efa2, nleF, nleH1-2, ecs1763, espM2, espW, espX1, nleH1-1, espR1, and espJ (OI-encoded)*.

### Serotyping and genotyping of flagellar (*fliC*) genes

Typing of O (lipopolysaccharide) and H (flagellar) antigens was performed as described (Beutin et al., 2004). Nonmotile (NM) *E. coli* strains were analyzed for their flagellar (*fliC*) genotypes by PCR and restriction fragment length polymorphisms (RFLP) of *Hha*I-digested *fliC* PCR products (Beutin et al., 2005). *fliC*-genotypes were confirmed by real-time PCR microarray testing as previously described (Bugarel et al., 2010a).

### Phenotypes

Stx production was determined using the Vero cell cytotoxicity assay and a commercially obtainable Stx enzyme immunoassay (Ridascreen-EIA, R-Biopharm AG, Darmstadt, Germany) as described previously (Beutin et al., 2007). Detection of the enterohemolysin or α-hemolysin phenotype of bacteria was performed on washed sheep blood (enterohemolysin) agar (Beutin et al., 2004). Sorbitol fermentation was tested on Sorbitol MacConkey (SMAC) agar, and B-D-glucuronidase activity on Fluorocult® E. coli 0157:H7 agar. Tellurite resistance was evidenced by the ability of strains to grow on cefixime-tellurite (CT)–SMAC agar and CHROMagar STEC. Media were obtained from Merck KGaA, Darmstadt, Germany, and CHROMagar, Paris, France.

### Detection of single and multiple copies of *stx* genes and subtyping

All isolates were screened by PCR for the presence of *stx*1 and *stx*2 using common primers (Beutin et al., 2007). *stx* genes were subtyped using PCR/RFLP analysis or subtype-specific primers as described (Beutin et al., 2007). The PCR/RFLP approaches allow to discriminate between *stx*1a (formerly *stx*1), *stx*1c, and *stx*1d genes as well as between *stx*2a (formerly *stx*2), *stx*2c/stx2d (variants *stx*2v-ha, *stx*2v-hb, *stx*2-NV206, *stx*2-EC1586, *stx*2d-O73), and stx2g genes according to differences in their B-subunits. The PCR/RFLP method has the advantage that strains carrying multiple variants of the *stx*2 gene can be identified. Toxin genotypes *stx*2b (formerly *stx*2-O118 and *stx*2-OX3:H21), *stx*2e, and *stx*2f were detected by PCR using subtype-specific primers as described (Beutin et al., 2007). Controls used were C600(H19) *stx*1a (GenBank accession no. M16625), DG131/3 *stx*1c (Z36901), and MHI813 *stx*1d (AY17085), C600(W34) *stx*2a (X07865), CB2851 *stx*2v-ha (AJ605767), CB7753 *stx*2v-hb (AF479829), NV206 *stx*2-NV206 (AF329817), RL481/06 *stx*2-EC1586 (AM498375), C165-02 *stx*2d-O73 (DQ059012), 12422 *stx*2g (AY286000), EH250 *stx*2b (AF 043627), 3615-99 *stx*2e (AJ313016), and T4/97 *stx*2f (AJ0110730).

### Detection of the mucus-activatable stx2 variant gene *stx*2d

All STEC strains were tested by PCR for the presence of the mucus-activatable *stx*2d using primers stx2d-F1, stx2d-R1, stx2d-R2, and stx2d-O55-R and following the new subtyping protocol developed by (Scheutz et al., 2012). In order to confirm the presence of *stx*2d and to further characterize the strains, we sequenced the *stx*2AB genes of all *stx*2d-positive and selected *stx*2d-negative strains (CB11017, 96/00 [CB12249], FP023 [CB12251], FP-087 [CB12260], I-005 [CB12275], 276-X [CB12282], 377-11 [CB12283], and C11 [CB12285]). Briefly, these strains were used for further PCRs with primers SLT-II-vc and CKS2, which flank the *Pst*I site in the A-subunit and the complete B-subunit of the Stx2 toxin (Jelacic et al., 2003) as well as primers stx2all-f (CGGGCCTTTTTTAATCTGCG) and stx2all-r (ACCCACATACCACGAATCAG), which flank the complete stx2AB genes (this work). The resulting 890 bp-amplicons and 1334 bp-amplicons, respectively, were purified and sequenced at a DNA sequencing service unit (Eurofins MWG Operon, Ebersberg, Germany). The sequences were analyzed using Lasergene software (DNASTAR, Madison, WI) and Accelrys Gene software (Accelrys Inc., San Diego, CA). Using the Blast algorithmus, the sequences were compared with sequences deposited at NCBI (http://www.ncbi.nlm.nih.gov/blast/). The following sequences were used as references: *stx*2a (accession no X07865), *stx*2b (AF043627), *stx*2c (M59432), and *stx*2d (AF479829).

### Nucleotide sequence accession number

The nucleotide sequences obtained have been entered into the EMBL database under accession numbers FR846369.1 to FR846379.1, and FR850037.1.

### Accessory virulence genes

The presence of accessory virulence genes was determined by a real-time PCR microarray as previously described (Bugarel et al., 2010a). Strains were screened for the presence of 42 genes associated with human virulent STEC (enterohemorrhagic *E. coli* [EHEC]) strains (Table 2). These genes were the LEE-encoded *eae* gene encoding intimin, six non-LEE-encoded effector (*nle*) genes issued from OI-122 (*pagC, ent/espL2, nleB, nleE*, *efa1*, *efa2*), six genes issued from OI-71 (*nleF, nleH1-2, nleA, nleG, espM1, ecs1822)* which mainly encode translocated substrates of the type III secretion system, as well as three genes issued from OI-43/OI-48 (*iha*, *ureD* [marker for the *ure* gene cluster], *terE* [marker for the *ter* gene cluster]) which encode the iron-regulated gene homolog adhesin Iha, urease activity and tellurite resistance, respectively. The array was also designed for the detection of seven genes derived from EHEC O157 virulence plasmid pO157 and STEC O113 viulence plasmid pO113, respectively: *ehxA* (enterohemolysin), *espP* (extracellular STEC-specific serine protease), *katP* (catalase peroxidase), *etpD* (the type II secretion system), *toxB* (putative EHEC adhesin), *saa* (autoagglutinating adhesin), and *subA* (subtilase cytotoxin). Other putative virulence factors, such as long polar fimbriae (*lpfA_O157_, lpfA_O113_*) and a transcriptional regulator of enteroaggregative *Escherichia coli* (*aggr*) were additionally screened.

**Table 2:** ***E. coli* gene targets for the real-time PCR micro array**.

**Gene (ORF name if chromosomal)**	**Encoded protein or family effector**	**Genetic support (mobile elements)**
*pag*C (Z4321)	PagC-like membrane protein	OI-122^a^
*ent* (Z4326)	Ankyrin repeats	OI-122^a^
*nleB* (Z4328)	Non-LEE-encoded type III effector	OI-122^a^
*nleE* (Z4329)	NleE	OI-122^a^
*efa1* (Z4332)	EHEC factor for adherence	OI-122^a^
*efa2* (Z4333)	EHEC factor for adherence	OI-122^a^
*nleF* (Z6020)	Non-LEE-encoded type III effector	OI-71^a^
*nleH1-2* (Z6021)	Non-LEE-encoded type III effector	OI-71^a^
*nleA* (Z6024)	Non-LEE-encoded type III effector	OI-71^a^
*nleG* (Z6010)	NleG	OI-71^a^
*espM1* (Z2565)	Non-LEE-encoded type III effector	OI-71^a^
*ecs1822 (Z6011)*	Hypothetical protein	OI-71^a^
*ureD* (Z1142)	Urease-associated protein UreD	OI-43^a^ & OI-48^a^
*terE* (Z1176)	Tellurite resistance cluster	OI-43^a^ & OI-48^a^
*Iha (Z1148)*	iron regulated gene A homolog adhesin	OI-43^a^ & OI-48^a^
*espX1 (Z0025)*	Pentapetide repeats	OI-1^a^
*espY1 (Z0065)*	SopD N-terminal domain	
*espY3* (Z0521)	Non-LEE-encoded type III effector	OI-26^a^
*nleH1-1* (Z0989)	NleH	OI-36^a^
*nleD-2* (Z0990)	NleD	OI-36^a^
*espX2 (Z1019)*	Non-LEE-encoded type III effector	OI-37^a^
*espV* (Z1387)	AvrA	OI-44^a^
*espK* (Z1829)	Leucine-rich repeats	OI-50 (prophage CP-933N)^a^
*espX7* (Z1822)	non-LEE-encoded type III effector	OI-50^a^
*espO1-1*	OspE family	OI-50^a^
*espN (Z1823)*	CNF	OI-50^a^
*ecs1763 (Z2040)*	Hypothetical protein	OI-57^a^
*espR1 (Z2242)*	Leucine-rich repeats	OI-62^a^
*espJ2 (Z3071)*	EspJ	OI-79^a^
*espM2* (Z3918)	IpgB	OI-108^a^
*espW* (Z3920)	HopW	OI-108^a^
*lpfA (Z4971)*	Long polar Fimbrial protein LpfA	OI-141 & OI-154^a^
*eae* (Z5110)	intimin	LEE^a^
*espG (Z5142)*	EspG	LEE^a^
*espF1 (Z5100)*	EspF (secreted effector)	LEE^a^
*kat*P	Catalase peroxidase	EHEC-plasmid^b^
*tox*B	Adhesin	EHEC-plasmid^b^
*etp*D	Type II effector	EHEC-plasmid^b^
*ehx*A	Enterohemolysin	EHEC-plasmid^b,c^
*esp*P	Serine protease EspP	EHEC-plasmid^b,c^
*saa*	STEC autoagglunating adhesin Saa	aEHEC-plasmid^c^
*subAB*	Subtilase cytotoxin SubAB	aEHEC-plasmid^c^ or putative PAI

a *Nomenclature of ORFs refers to sequence of E. coli O157:H7 EDL933 (GenBank AE005174)*.

b *Plasmid pO157 EDL933 (Genbank NC_007414)*.

c *Plasmid pO113 (GenBank NC_007365); PAI, pathogenicity island*.

Furthermore, all strains were tested by PCR for the presence of the *espI* gene encoding a serine protease, termed EsPI, that is located in the chromosome on the locus of protease and adherence (LPA). Primers *espI*-I and *espI*-II and cycling conditions are used as previously described (Schmidt et al., 2001).

All *sub*A-positive strains were tested by PCR for the presence of the *tia* gene (Genbank accession no. U20318) using primers tia_lo and tia_sense as described by Tozzoli et al. (2010). *tia* encodes for an invasion determinant described in enterotoxigenic *E. coli*.

### PFGE

Pulsed-field gel electrophoresis was performed using the standardized PulseNet protocol published previously (Gerner-Smidt and Scheutz, 2006). Briefly, agarose-embedded DNA was digested with 50 U of *Xba*I (Roche Diagnostics GmbH, Mannheim, Germany) for 4 h at 37°C. The restriction fragments were separated by electrophoresis in 0.5 × Tris-borate-EDTA buffer containing 50 μM thiourea at 14°C for 19 h by the use of a CHEF DR-III system (Bio-Rad, Munich, Germany), with pulse times of 2.16 to 54.17 s. *Xba*I-digested DNA of *Salmonella enterica* serovar Braenderup strain H9812 (Centers for Disease Control and Prevention, Atlanta, GA) was used as a molecular size marker. Similarities between restriction patterns were calculated with BioNumerics software (version 6.6, Applied Maths, Ghent, Belgium) using the Dice coefficient with band matching parameters of 1.0% optimization and 1.5% position tolerance. Interstrain relationships were assessed by cluster analysis using the Unweighted Pair-Group with Mathematical Average (UPGMA) method.

## Results

### Two serotypes dominate among stec O178 strains

Sixty four O178 strains were motile and could thus be serotyped for their H antigens. Among them were 61 O178:H19 and each one O178:H7, O178:H10, and O178:H16 strains. Ten strains were nonmotile and the flagellar types were determined by *fliC* genotyping. Five of these were O178:[H19] and five O178:[H7]. In total, 66 of the strains were serotyped as O178:H19, six as O178:H7 and each one as O178:H10 and O178:H16 (Table 1).

### Phenotypes

Cytotoxic activity on Vero cells and production of Stx tested by an enzyme immunoassay was found with all 66 O178:H19, four O178:H7 and the O178:H10 strain(s). Two O178:H7 strains and the O178:H16 strain were Stx-negative with both methods. All strains possessing the *ehxA* gene produced enterohemolysin. No strain produced α-hemolysin. All strains fermented sorbitol and show B-D-glucuronidase activity. All strains possessing the *terE* gene grew on CT–SMAC agar and CHROMagar STEC.

### Association between *stx*-genotypes and *E. coli* O178 serotypes

All but two solely *stx*1 positive O178 STEC strains (2.8%) carried *stx*2 genes (97.2%) and 33 strains of which (46.5%) carried both *stx*1 and *stx*2 genes (Table 1). All 30 *stx*1 positive O178:H19 strains and the single O178:H10 strain carried the *stx*1a subtype. The *stx*1c subtype was present in all four STEC O178:H7 strains (Table 1).

Thirty-one (47.0%) of the 66 STEC O178:H19 strains carried the *stx*2a gene, 32 (48.5%) the *stx*2c gene and eight (12.1%) the *stx*2d gene. Except in one case, combined presence of *stx*2a and *stx*2c was not observed, but the *stx*2a and the *stx*2d genes were present together in four (6.1%) of the 66 STEC O178:H19 strains. The *stx*2b gene was found in one (1.5%) of the 66 STEC O178:H19, but in three (75.0%) of the four STEC O178:H7 strains (Table 1). Other *stx*2-subtypes were not detected in any of the strains.

### Detection of mucus-activatable *stx2d* by PCR and nucleotide sequencing

**Figure 1:**
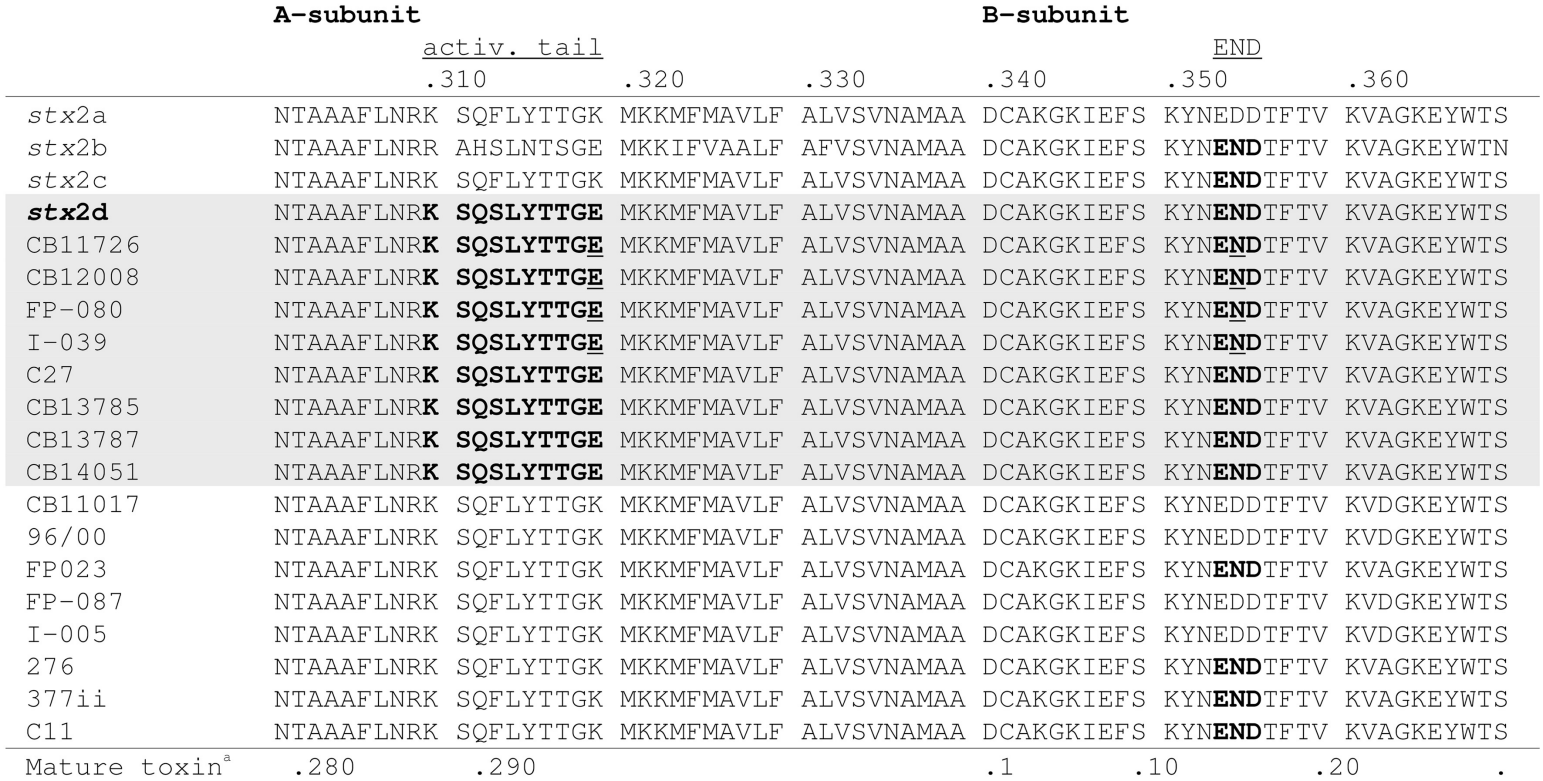
**Alignment of the amino acid sequences of the C-terminal ends of the A-subunits and B-subunits of 8 *stx*2d-positive and 8 *stx*2d-negative O178:H19 strains investigated in this study**. Amino acid sequences in bold show the activatable tail in the A-subunit and the END motif in the B-subunit. Amino acid sequences which contain both motifs, thus correspond to the mucus-activatable *stx*2d variant, are shaded. The following sequences were used as references: *stx*2a (GenBank accession no. X07865), *stx*2b (AF043627), *stx*2c (M59432), and *stx*2d (AF479829). Amino acid sequences underlined indicate double peaks in the chromatogram which reveal multiple *stx*2-toxin types in these strains. ^a^Position in mature toxin sequence.

By PCR eight of the STEC O178:H19 strains (12.1%) were identified as *stx*2d-positive. The STEC O178:H7 and O178:H10 strains were found negative for *stx*2d (Table 1). To confirm the results, we determined the nucleotide sequences of the *stx*2AB genes of all *stx*2d-positive and 8 selected stx2d-negative STEC O178:H19 strains. The sequences were analyzed and compared with the published *stx*2-reference sequences in GenBank. It is noteworthy, that chromatograms derived from STEC strains carrying both *stx*2a and *stx*2d genes showed overlapping peaks at nucleotide positions which differ between *stx*2a and *stx*2d (data not shown). The sequences were translated into putative amino acid sequences of the A and B subunits and analyzed for the activatable type of a Stx2 toxin. Scheutz et al. (2012) defined the activatable type of a Stx2 toxin as the combination of the “activatable tail” in the Stx2A subunit and the END motif in the Stx2B subunit The activatable tail consists of the last 10 amino acids in the C-terminal end of the A2 subunit and has been identified as KSQSLYTTGE from position 288 to 297 in the mature toxin sequence (with the relevant amino acids serine at position 291 and glutamic acid at the final position 297); the motif END is found at position 14 to 16 in the B subunit. Only the Stx sequences of the eight O178:H19 strains CB11726, CB12008, FP-080 [CB12258], I-039 [CB12278], C27 [CB12288], CB13785, CB13787 and CB14051 tested as *stx*2d-positive by PCR, fulfilled these criteria, and are therefore considered to be activatable. The other strains revealed neither the activatable tail in the A subunit nor the END motif in the B subunit or only one of these characteristic traits (Figure 1).

### Presence of accessory virulence-associated genes

Twenty eight of the LEE- or OI-encoded virulence-associated genes (eae, *ent/espL2*, *nleB*, *nleE*, *efa1*, *efa2, nleF, nleH1*-*2, nleA, espG, ecs1763, espM1*, espN, *espM2*, *espX2, espX7, espO1-1, espY4-2, espX6*, espW, espY1, espY3, *nleH1*-*1*, *nleD-2, nleG*, *espJ*, *ecs1822*, and *ureD*) were not present in any of the O178 strains investigated. All O178 strains were also tested negative for the pO157-encoded *katP, etpD* and *toxB* genes, as well as the *lpfA_O157_* and *aggr* genes. An exception was the Stx-negative O178:H7 strain CB09017 from a human patient which was positive for the eae and for several nle genes (*ent*/*espL2*, *nleB*, *nleE*, *efa1*, *efa2, nleF, nleH1*-*2, espG*, *espF1, ecs1763, espM2*, espW, *espX1, nleH1*-*1, espR1*, and *espJ*). According to its virulence profile and the absence of the *bfp* gene encoding bundle-forming pili (data not shown), CB09017 was classified as an atypical EPEC strain. The LPA-encoded *espI* was not found in any of the *E. coli* O178:H19, O178:H10, and O178:H16 strains, but was detectable in four O178:H7 strains. Genes *iha* and *espP* were common in all STEC O178 strains, but were missing in the three Stx-negative strains. Characteristic traits and (putative) virulence genes detected in the O178 strains are listed in Table 1. *pagC* was detected in 36 of the O178:H19 strains (54.5%) and 31 (47.0%) were associated with PFGE cluster B, C, D and E strains (see below). *iha* which encodes the iron-regulated gene homolog adhesin IrgA was found in all O178:H19 strains, whereas *terE* which encodes tellurite resistance was detected in 16 O178:H19 strains (24.2%). Except for FP-080, in all STEC O178 strains *lpfA_O113_* which encodes the major subunit of LPF of STEC O113 was detectable. Among the seven virulence genes derived from virulence plasmids, *espP* was found in all O178:H19 strains; *ehxA* and *saa* were detected each in 35 O178:H19 strains (53.0%), whereas *subAB* was present in 18 O178:H19 strains (27.3%) which belonged all to PFGE cluster A (see below). All *subAB*-positive O178:H19 strains were *tia*-negative. The four O178:H7 STEC strains isolated from human patients and from deer meat harbored *ehxA, espP, subA, iha*, in addition two of them harbored *terE*. In contrast to O178:H19 strains, they all were also *tia*- and *espI*-positive. The *stx*-negative O178:H7 strain isolated from cattle and the *stx*-negative O178:H16 strain isolated from salad carried none of the virulence genes investigated (except *lpfA_O113_*). The human EPEC O178:H7 strain did not harbor genes other than the *eae* and *nle* genes mentioned above. The O178:H10 STEC strain from deer meat carried *espP, subAB*, and *iha* genes, but no *tia* gene. None of the O178:nonH19-strains possessed *pagC* and *saa* genes (Table 1).

### PFGE genotyping of STEC O178:H19 strains

**Figure 2:**
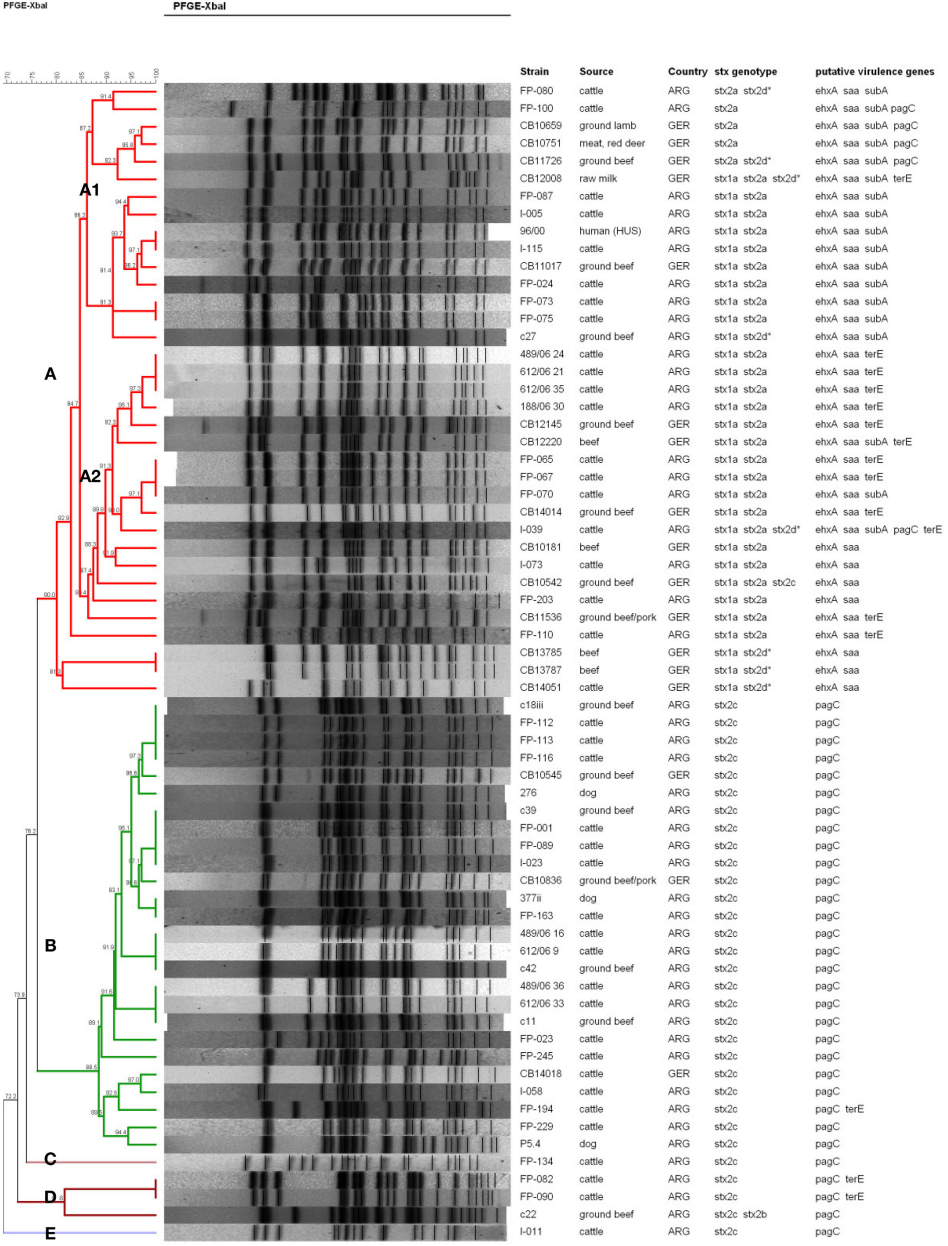
**PFGE-based clustering of 66 STEC O178:H19 strains investigated in this study**. Clusters A–E were defined using a cut-off value of 80% similarity and are indicated by different colors. The degree of similarity (%) is shown on the scale at the top left of the figure. *stx*2d*, mucus-activatable *stx*2d. All STEC O178:H19 strains possessed *espP* and *iha*, and all except strain FP-080 *lpfA*_O113_. None of the STEC O178:H19 strains possessed *katP, etpD*, and *toxB*.

The genetic relatedness between the different *E. coli* O178 strains was investigated by *Xba*I-PFGE genotyping. *E. coli* O178 strains carrying other H-antigens/*fliC* types than H19 revealed individual PFGE patterns and proved to be genetically more distant (data not shown). The 66 O178:H19 strains revealed 47 different PFGE patterns. A dendrogram based on similarities of the PFGE patterns was created with the BioNumerics software v. 6.6 using the band-based Dice coefficient (Figure 2). Sixty-one of the O178:H19 strains were assigned to two major PFGE clusters designated A (35 strains) and B (26 strains) (Figure 2). The five remaining strains were assigned to clusters C (one strain), D (three strains), and E (one strain).

Clusters A to E were defined using a cut-off value of 80% similarity (Figure 2). Strains belonging to cluster A and B, respectively, show only 76.2% similarity in their PFGE patterns and also clearly differ in their virulence traits. Cluster A-O178:H19 STEC (>80.0% similarity) carry the pO113 virulence plasmid-genes *ehxA, saa* and *espP* together with the *stx*2a and/or *stx*2d, *iha* and *lpfA_O113_* genes. Thirty cluster A-strains additionally carry the *stx*1a gene. Cluster A was further subdivided into subclusters A1 (15 strains, 86.2% similarity) and A2 (16 strains, 86.4% similarity) and, more distantly related, four additional strains. Subcluster A1-strains were additionally characterized by presence of the pO113-encoded *subAB* gene which was only found in three of the 15 subcluster A2-strains. Nearly 70% of the subcluster A2-strains carry an additional *terE* gene which was not detected in A1-strains (except in strain CB12008). The genetically highly related subclusters A1 and A2 encompassed both strains from Argentina and Germany. The STEC O178:H19 HUS-patient isolate from Argentina (96/00) grouped within subcluster A1 together with four isolates from Argentinean cattle and one isolate from German ground beef with a high degree of similarity (93.7%). The HUS strain 96/00 was found 100% similar to the strain I-115 from Argentine cattle and 97.1% similar to the German food strain CB11017.

The 26 cluster B-O178:H19 strains (88.5% similarity) were from cattle, beef, and from dogs, only three of these strains were from Germany. Cluster B-strains were characterized by the *stx*2c gene: the *stx2v-ha* variant was present in 22 strains (84.6%) and *stx2v-hb* in 4 strains (15.4%). Characteristically, all strains were also positive for *pagC*, *lpfA_O113_*, *iha*, and *espP*, and were, in contrast to cluster A–strains, negative for *stx*1a, *ehxA*, *saa*, and *subAB*. Cluster B contains strains of different sources and geographical origins with high similarities up to 100%. Among them are strains from three Argentine dogs which show high similarity (94.4 – 100%) to strains from cattle and food of bovine origin.

## Discussion

Over the past years, STEC O178:H19 became one of the most prevalent serotypes isolated from dairy cows (Fernandez et al., 2010) and beef cattle (Tanaro et al., 2012) as well as in beef abattoirs (Masana et al., 2011) and in food of bovine origin in Argentina and Germany (Beutin et al., 2007; Sanz et al., 2007; Werber et al., 2008; Slanec et al., 2009; Kruger et al., 2011). Moreover, this serotype was prevalent in pets (Bentancor et al., 2012). In Argentina where the incidence of HUS is high, STEC O178:H19 has been already isolated from a human patient with HUS (Giugno et al., 2007). In Germany, some sporadic cases of human infections with STEC O178:H19 were reported (Bielaszewska et al., 2006; Werber et al., 2008). There are several reasons why STEC O178:H19 strains might represent an “emerging” pathogen. On one hand, the O178 serogroup was defined quite recently and was not part of the *E*. *coli* serotyping scheme before 2004 (Scheutz et al., 2004), and even today complete serotyping is rarely performed in routine investigations. On the other hand, modified cattle feeding management practices in Argentina is conceivable as another reason for the “emergence” of STEC O178 strains. The intensive feeding and high animal density in feedlot could give rise to a selective bacterial flora with *E. coli* serotypes that differ from those isolated previously in grazing-fed cattle (Parma et al., 2000; Padola et al., 2004).

The aim of this study was to characterize and to assess the potential risk of STEC O178 strains isolated from different sources for humans. Our findings indicate that STEC O178 strains lack typical properties that are closely associated with enterohemorrhagic *E. coli* (EHEC) strains that are frequently isolated from outbreaks and from human HC and HUS cases. STEC O178 strains were negative for the LEE-encoded *eae* gene as well as for non-LEE effector genes encoded by O-islands 122, 71 and 57 which have been identified as markers related to potentially highly virulent STEC (Karmali et al., 2003; Coombes et al., 2008; Konczy et al., 2008; Bugarel et al., 2010b). As an exception, a *pagC*-like gene encoding a putative PagC-like outer membrane adhesin is present in 36 (54.5%) of the O178:H19 isolates. This gene was shown to reside on the first of three modules composing OI-122 in the EDL933 genome (Konczy et al., 2008). However, in the LEE-negative O113:H21 strain CL3, *pagC* is not part of an OI-122-like structure, but rather a part of a unique hybrid genomic island containing segments of OIs 122 and 48 (Shen et al., 2004). Sequence analysis of the *pagC*-like gene in LEE-positive and LEE-negative STEC strains revealed significant differences in their composition (Konczy et al., 2008). LEE-positive *pagC*-strains always possessed module 2 and/or module 3 besides the *pagC*-module 1. LEE-negative *pagC*-strains always possessed exclusively the *pagC*-module 1 and differed in their *pagC* alleles from LEE-positive strains. LEE-positive *pagC*-strains were assigned to highly virulent human seropathotype A, B, or C strains, and LEE-negative *pagC*-strains were grouped either in seropathotype C (human strains) or seropathotypes D and E (bovine strains) with low or no virulence to humans (Karmali et al., 2003; Konczy et al., 2008). Accordingly, the O178:H19 *pagC*-strains from this study might be classified as seropathotype D or E strains at this point. The large majority of our *pagC*-strains are grouped in PFGE-cluster B with a high degree of similarity and carry a limited repertoire of virulence genes (*stx*2c, *pagC*, *lpfA_O113_*, *iha*, and *espP*) (Figure 2).

However, the presence of LEE- and *nle*-genes is not essential for STEC pathogenesis and sporadic cases and small outbreaks of STEC infections, including HC and HUS, have been caused by LEE-negative strains (Keskimaki et al., 1997; Bonnet et al., 1998; Paton et al., 1999). In the absence of LEE, mechanisms are emerging by which the LEE-negative STEC interact with the host intestinal mucosa and induce disease (Newton et al., 2009). Recently, a number of putative adhesins have been identified, such as the Long Polar Fimbrial Protein LPFA_O113_, the STEC autoagglutinating adhesin (Saa), and an iron-regulated gene homolog adhesin (Iha) (Paton et al., 1999; Tarr et al., 2000; Doughty et al., 2002).

The *lpfA_O113_* gene, located within a novel fimbrial gene cluster, was initially reported in a LEE-negative EHEC O113:H21 strain isolated from a HUS patient. It was shown that deletion of the fimbrial subunit gene *lpfA_O113_* resulted in decreased adherence of these bacteria to epithelial cells *in vitro* (Doughty et al., 2002). Remarkable, *lpfA_O113_* was also found in other LEE-negative EHEC and in non-O157:H7 LEE-positive EHEC strains (Doughty et al., 2002).

Saa was first characterized from a LEE-negative O113:H21 STEC strain responsible for an outbreak of HUS (Paton et al., 1999). Over the past years the Saa-encoding gene has been detected in many LEE-negative STEC strains from animals, food and humans (Zweifel et al., 2004, 2005; Cergole-Novella et al., 2007; Oliveira et al., 2007, 2008; Vu-Khac and Cornick, 2008; Fernandez et al., 2010; Bandyopadhyay et al., 2011; Bosilevac and Koohmaraie, 2011) including samples from HUS patients (Paton et al., 2001; Paton and Paton, 2002; Jenkins et al., 2003). The *saa*-positive STEC O178:H19 isolates from our study belonged all to PFGE-cluster A which comprises O178:H19 strains with a high virulence potential. The *saa*-positive STEC isolates (47.3%) were all *ehxA*-positive, too, since both genes are encoded in the pO113 megaplasmid (Newton et al., 2009). By contrast, the *saa* and *eae* genes appear to be mutually exclusive within STEC strains, so that *saa* has been suggested to serve as an alternative adhesin of LEE-negative strains in human colonization. The *saa* gene from different STEC strains vary in size due to the number of direct repeated units at the 3′ end, and five *saa*-variants were identified until now (Paton et al., 2001; Lucchesi et al., 2006). However, binding properties of *saa*-carrying STEC to epithelial cells exhibit marked variation with no correlation to the number of C-terminal repeats and to the Saa-expression (Toma et al., 2008). Clarification of the role of *saa* in human infections needs further investigation, however, its high prevalence in bovine and ovine STEC suggest a role in the attachment to bovine and ovine gut (Jenkins et al., 2003; Zweifel et al., 2004; Fernandez et al., 2010; Tanaro et al., 2012).

Pathogenicity islands OI-43 and OI-48 are duplicate genomic islands present in EHEC O157:H7 strains. OI-43/48 carry three functional gene clusters, encoding urease (*ure* cluster), tellurite resistance (*ter* cluster), and putative adhesins Iha (*iha*) and AIDA-1 *(aidA-1*). Iha similar too Saa, was suggested to serve as adhesin in LEE-negative STEC strains. The role of Iha in human virulence is not clear yet, as it was found in similar frequencies in STEC seropathotypes highly associated with severe diseases as well as in less or apathogenic strains (Ju et al., 2013). It is noteworthy, that *iha* has different origins. Besides the OI-43/48 origin, it was also found in plasmid pO113 of LEE-negative O113:H21 (Newton et al., 2009) as well as on the newly described genomic island LPA in STEC O91:H- (Schmidt et al., 2001). In our study *iha* is present in all STEC O178 strains, but is missing in the three Stx-negative strains. In contrast to *iha*, OI-43/48 encoded *ure* and *ter* genes were closely associated with seropathotypes causing severe disease and outbreaks (Ju et al., 2013). Urease helps STEC to survive in the stomach and amends its ability to colonize the calf and human intestinal tracts. The role of tellurite resistance genes is not completely clear. However, it was postulated that *ter* genes might encode an adhesin-related gene product and/or might offer a selective advantage in the host environment and under stress conditions (Yin et al., 2009). No O178 strain in this study was *ureD*-positive, 24.3% of the strains were *terE*-positive. Tellurite resistance of these strains was evidenced by their ability to grow on CT-SMAC agar and CHROMagar STEC (data not shown).

Among other toxins a novel subtilase cytotoxin (SubAB) has been reported to contribute to the virulence and pathogenicity of LEE-negative STEC for humans. SubAB is the prototype of a new AB_5_ toxin family first detected in the LEE-negative O113:H21 STEC strain 98NK2 that caused an outbreak of HUS in Australia in 1998 (Paton et al., 2004). Since then the *subAB* operon has been detected in STEC isolates from different continents belonging to over 30 STEC serogroups (Irino et al., 2010; Paton and Paton, 2010). In Belgium SubAB was found in one O178:H19 HUS-associated strain (Buvens et al., 2010). SubAB is lethal for mice and induces pathological traits overlapping those seen in the HUS. It was suggested (Paton and Paton, 2010) that SubAB directly contributes to pathology in humans infected with STEC strains that produce both Stx and SubAB. The question, whether the production of SubAB correlates to the severity of STEC disease in humans needs further investigation. In our study, nearly 30% of the STEC O178:H19 strains carry *subA*, in all cases together with the *saa*, *ehxA*, and *espP* genes, suggesting their location on the pO113 plasmid as found with STEC O113:H21 (Newton et al., 2009) (Table 1). It is noteworthy, that all *subAB*-carrying STEC O178:H19 strains isolated in Germany and Argentina, among them the Argentinian HUS strain, group in the same PFGE-subcluster A1 indicating their genetic similarity (Figure 2). Argentina has a very high incidence of STEC infections and HUS cases, many of them caused by LEE-negative, but SubAB-positive STEC strains (Gerhardt et al., 2009). In future, SubAB-producing STEC strains should be payed more attention with regard to their role to cause or to augment STEC diseases. It has been suggested that different variants of *subAB* exist (Tozzoli et al., 2010; Orden et al., 2011). One called *SubAB1* is encoded by the pO113 megaplasmid as found in the human pathogenic STEC O113:H21 98NK2 prototype strain. The other, called *SubAB2*, is chromosomally located close the *tia* gene (an invasion gene previously described in enterotoxigenic *E. coli)* on a *subAB-tia* putative pathogenicity island (Tozzoli et al., 2010; Orden et al., 2011; Michelacci et al., 2013). Virulence features of the SubAB-positive STEC O178:H19 strains in our study (*saa*-positive, *tia*-negative) suggest that they harbor the *SubAB1* variant typical associated with bovine strains (Table 1). By contrast, the SubAB-positive STEC O178:H7 strains isolated from human patients and from deer meat revealed the *stx* genotype *stx*1c, *stx*2b and were *tia*-positive and *saa*-negative, suggesting the presence of *subAB2* (Table 1). Further work is needed, to explore the role of SubAB toxins in severe disease.

The role of *stx*2a and *stx2*d genes as major virulence factors in STEC infections of humans was previously investigated (Friedrich et al., 2002; Persson et al., 2007; Scheutz et al., 2012). The mucus-activatable Stx2d was found in LEE-negative STEC causing severe disease including HC and HUS in humans (Melton-Celsa et al., 1996, 1998, 2002; Paton et al., 1999; Jelacic et al., 2003; Bielaszewska et al., 2006). Strains producing Stx2d proved to be highly virulent in experimental infections of streptomycin-treated mice (Melton-Celsa et al., 1998). Epidemiological and toxicity data point to a high pathogenic potential of Stx2d-producing STEC for humans. At present, limited data are available regarding the occurrence of strains harboring the *stx*2d gene in cattle, other livestock sources and foods (Gobius et al., 2003; Oliveira et al., 2008; Zheng et al., 2008; Slanec et al., 2009; Xia et al., 2010). In our study, we used a recently developed PCR (Scheutz et al., 2012) to identify the *stx*2d gene in 8 of the 74 STEC O178 strains (10.8%) investigated in this study. The PCR results were confirmed by nucleotide sequencing of Stx2 genes. Like Stx2a-strains, Stx2d-strains were exclusively found in PFGE-cluster A.

Generally, serogroup O178 belongs to those serogroups of *E. coli* that covers multiple lineages of STEC strains which express different H-types (Scheutz and Strockbine, 2005). Strains which express H10, H12, H16, H21, and H27 are very rare and often avirulent. O178:H7 strains are heterogeneous and include STEC, EPEC and avirulent strains. A particular H7-lineage has emerged that substantially differ from the O178:H19 lineage. Strains of this lineage analyzed in this study originate from deer and humans and are associated with the genotype *stx*1c/*stx*2b/*ehxA*/*subAB2*/*espI*/*[terE]*/*espP*/*iha*.

However, in this study we have focused on STEC O178:H19 strains which emerged in numerous countries worldwide. STEC O178:H19 strains are heterogeneous and split up into distinct PFGE-clusters of related strains. The two major PFGE-clusters A and B differ in their *stx*-genotypes and non-Stx putative virulence traits, including adhesins, toxins, and serine-proteases. PFGE-cluster B includes strains of low virulence attributed to the very limited spectrum of virulence genes (*stx*2c, *pagC*, *lpfA_O113_*, *espP*, *iha*). By contrast, PFGE cluster A comprises more virulent strains carrying virulence genes related to severe clinical outcomes (*stx*2a, *stx*2d, *ehxA*, *saa*, *subAB1*, *lpfA_O113_*, *terE* combined with *stx*1a, *espP*, *iha*). The toxic *stx*2a, the mucus-activatable *stx*2d, the enterohemolysin gene *ehxA*, and the novel adhesin/cytotoxin genes *lpfA_O113_, saa*, and *subAB* might enhance the pathogenicity of the strains, enabling them to adhere to the intestinal epithelium and to produce a potentially dangerous toxin. Such strains are an increasing risk for humans, because they, unlike other LEE-negative strains, may cause human diseases. Therefore, the risk represented by them to public health should be carefully monitored.

### Conflict of interest statement

The authors declare that the research was conducted in the absence of any commercial or financial relationships that could be construed as a potential conflict of interest.
